# Ethylene participates in strigolactone-triggered stomatal closure via Gα protein-activited hydrogen peroxide and hydrogen sulfide synthesis in *Arabidopsis*

**DOI:** 10.3389/fpls.2026.1796780

**Published:** 2026-03-12

**Authors:** Yinli Ma, Zhenyu Zhao, Yuwei Song, Sida Chai, Hongyu Zhao, Shuangshuang Liang

**Affiliations:** College of Life Sciences, Shanxi Normal University, Taiyuan, China

**Keywords:** ethylene, Gα protein, hydrogen peroxide, hydrogen sulfide, stomatal movement, strigolactones

## Abstract

The signaling cascade of strigolactone (SL)-regulated stomatal closure in *Arabidopsis thaliana* was investigated using pharmacological assays, fluorescence microscopy, spectrophotometry, gas chromatography, RT-PCR, and qRT-PCR. In wild-type plants, GR24 (a synthetic strigolactone analog)-induced stomatal closure was significantly inhibited by ethylene biosynthesis/perception inhibitors or Gα inhibitor pertussis toxin (PTX). GR24 obviously promoted closure in *eto1-1*, *cGα1* and *wGα1* mutants, but failed to do so in mutants *etr1-1*, *etr1-3*, *gpa1–1* and *gpa1-2*. GR24 also upregulated 1-aminocyclopropane-1-carboxylic acid (ACC) synthase (ACS) gene *ACS* and heterotrimeric G-protein α subunit 1 gene *GPA1* transcript levels, showing that both ethylene synthesis and Gα activation were required for SL-induced stomatal closure. Ethylene biosynthesis/perception inhibitors significantly suppressed GR24-induced hydrogen peroxide (H_2_O_2_) production, hydrogen sulfide (H_2_S) synthesis, and L-/D-cysteine desulfhydrase (L-/D-CDes) activity increase in wild-type plants. These responses occurred in *eto1–1* mutant but not in *etr1* mutants. ACC-induced stomatal closure, H_2_O_2_ and H_2_S synthesis, and L-/D-CDes activity increase were obviously abolished in *AtrbohD*, *AtrbohF*, *AtrbohD/F*, *Atl-cdes* and *Atd-cdes* mutants, and exogenous H_2_O_2_ or sodium hydrosulfide (NaHS) could significantly rescue the defect in *etr1* mutants, showing that ethylene acted via H_2_O_2_ and H_2_S synthesis in SL-induced stomatal closure. Furthermore, PTX obviously suppressed GR24-induced H_2_O_2_ and H_2_S synthesis, and L-/D-CDes increase in the wild type. These responses persisted in *cGα1* and *wGα1* mutants but were absent in *gpa1* mutants. Finally, cholera toxin (CTX)-induced stomatal closure and downstream responses required *AtrbohD/F* and *AtL-/D-CDe*s, and exogenous H_2_O_2_ and H_2_S rescued GR24-induced closure in *gpa1* mutants, suggesting that Gα activation acted upstream of H_2_O_2_ and H_2_S synthesis in SL-induced stomatal closure. In contrast, ACC failed to rescue the defect in GR24-induced stomatal closure treated with PTX in the wild type and in *gpa1* mutants. Notably, GR24 substantially increased ethylene production in both PTX-treated wild-type plants and in *gpa1* mutants, indicating that ethylene functioned by activating Gα in SL-induced stomatal closure. In summary, SL induced ethylene biosynthesis, which activated Gα. Activated Gα then promoted H_2_O_2_ production via AtrbohD/F and subsequent H_2_S synthesis via AtL-CDes/AtD-CDes, finally leading to stomatal closure.

## Introduction

As a class of plant hormones, strigolactones (SL) regulate numerous physiological processes of plants (Al-Babili and Bouwmeester, 2015). In *Arabidopsis thaliana*, SL biosynthesis follows a multi-step pathway that begins with carotenoids, involving key enzymes such as DWARF27 (D27), the carotenoid cleavage dioxygenases MORE AXILLARY GROWTH3 (MAX3) and MAX4, and the cytochrome P450 MAX1 (Waldie et al., 2014; Al-Babili and Bouwmeester, 2015). Notably, both SL and abscisic acid (ABA) are derived from carotenoid precursors and belong to the terpenoid lactone class of compounds (Matusova et al., 2005; López-Ráez et al., 2010). While widely recognized for regulating shoot architecture (Gomez-Roldan et al., 2008), SL also influence multiple developmental processes (Agusti et al., 2011; Rasmussen et al., 2012; Ueda and Kusaba, 2015) and participate in plant stress responses (Ha et al., 2014; Zhang et al., 2022). Mutants impaired in SL biosynthesis or signaling show increased susceptibility to drought, salt, and osmotic stress, indicating that SL contribute to stress adaptation (Ha et al., 2014; Liu et al., 2015). For example, in cotton, overexpression of SL biosynthesis genes *GhMAX3*/*GhMAX4b* or the receptor gene *GhD14* significantly enhances drought resistance (Dong et al., 2024, 2025). SL interact with hormones such as ABA, auxin, and cytokinin to coordinate abiotic stress responses (Bhoi et al., 2021). Zhao et al. (2025) found that SL negatively regulates vessel element formation, thereby affecting plant water transport and use efficiency; SL-dependent changes in vessel formation directly influence stomatal transpiration rates, highlighting the role of vascular tissue composition in water balance. Chen et al. (2024) demonstrated for the first time that SL are key regulators of root-associated microbial communities under varying phosphorus availability. Additionally, SL are involved in regulating stomatal movement (Lv et al., 2018; Ma et al., 2024). Lv et al. (2018) demonstrated that SL-induced stomatal closure depends on hydrogen peroxide (H_2_O_2_)/nitric oxide (NO) production and SLOW ANION CHANNEL-ASSOCIATED 1 (SLAC1) activation in an ABA-independent mechanism. Nevertheless, the precise mechanism by which SL regulate stomatal movement and integrate with other signaling pathways remains to be fully clarified.

Phytohormone interactions play a critical role in regulating numerous physiological processes and stress responses in plants (Wang and Irving, 2011; Daszkowska-Golec and Szarejko, 2013). Ethylene serves not only as a key regulator of plant growth and development but also as a central hub that integrates environmental signals—including mechanical stress, nutrient deficiency, drought, salinity, and wounding, and coordinates internal hormone networks, such as those involving ABA, jasmonic acid (JA), brassinosteroids (BRs), and auxin. For instance, Sevostyanova et al. (2025) demonstrated that ethylene sensitivity is essential for initiating adaptive hormonal reprogramming under salt stress, coordinating both stomatal closure and growth responses to alleviate stress effects. Ethylene can also upregulate the expression of *BRP*, a BR-repressed gene, in etiolated *Arabidopsis* seedlings (Gendron et al., 2008). Conversely, BRs promote ethylene biosynthesis by inducing ACS expression in *Vigna radiata* (Yi et al., 1999) or stabilizing ACS protein in *Arabidopsis thaliana* (Hansen et al., 2009). Additionally, ethylene acts downstream of BR signaling to enhance cotton fiber elongation (Shi et al., 2006). A recent study by An et al. (2025) revealed that ethylene upregulates the transcription and protein stability of the protein phosphatase 2C (PP2C) members ABI1 and ABI2 via Ethylene Insensitive 2 (*EIN2*)-*EIN3* signaling pathway, thereby inhibiting ABA-induced stomatal closure, which uncovers a novel molecular mechanism of ethylene-mediated antagonism toward ABA responses. Notably, research on ethylene’s effect on stomatal movement has produced inconsistent results. Some studies indicate that ethylene promotes stomatal opening (Levitt et al., 1987) and can inhibit stomatal closure induced by ABA or methyl jasmonate (MeJA) (Tanaka et al., 2005; Watkins et al., 2014; Munemasa et al., 2019). In contrast, other reports show that ethylene induces stomatal closure (Desikan et al., 2006; Liu et al., 2011). Ethylene has also been found to mediate UV-B-induced stomatal closure (He et al., 2011) and participate in BR-induced stomatal closure via Gα protein-activated H_2_O_2_ production and NO synthesis (Shi et al., 2015). However, whether ethylene is involved in SL-regulated stomatal movement remains to be investigated.

Heterotrimeric G proteins, composed of α, β, and γ subunits, play essential roles in transducing transmembrane signals in both plants and animals. Extracellular signals are initially perceived by plasma membrane-localized G-protein-coupled receptors (GPCRs), which then activate G proteins. This activation promotes GTP binding to the Gα subunit and its dissociation from the Gβγ dimer. Both Gα and Gβγ subsequently regulate downstream effector enzymes to generate second messengers, thereby relaying extracellular signals into the cell (Oldham and Hamm, 2008; Tuteja, 2009). In plants, G proteins have been shown to participate in diverse biological processes, including growth, development and immune responses (Warpeha et al., 2007; Okamoto et al., 2009; Nilson and Assmann, 2010; Tsugama et al., 2013; Zhang et al., 2021). They are also involved in responses to various environmental stresses, such as salinity, drought, and nutrient deficiency. For example, *Arabidopsis* G-protein β subunit 1 (AGB1) enhances salt tolerance by modulating Na^+^ transport from roots to shoots and promoting the Basic Leucine Zipper 17 (bZIP17)-mediated unfolded protein response in the endoplasmic reticulum (Cho, 2024; Yadav et al., 2024). In ABA signaling, AGB1 negatively regulates ABA responses by competitively binding to Mitogen-Activated Protein Kinase 3 (MPK3) and inhibiting the phosphorylation of VirE2-INTERACTING PROTEIN 1 (VIP1) (Xu et al., 2024). Stomatal movement is subject to precise regulation by G proteins. For instance, extracellular calmodulin (ExtCaM) triggers stomatal closure via a signaling cascade that includes heterotrimeric G protein activation, increased cytosolic Ca²^+^ levels, and H_2_O_2_ production (Chen et al., 2004). The loss of GPA1 disrupts ABA signaling downstream of ABA perception but upstream of reactive oxygen species (ROS) generation (Zhang et al., 2011). Furthermore, Gα protein-activated H_2_O_2_ and NO generation mediate brassinosteroid (BR)-induced stomatal closure (Shi et al., 2015). More recently, Guo et al. (2025) demonstrated that G-protein α subunit 1 in *Arabidopsis* (GPA1) modulates stomatal opening and closing by binding to and inhibiting the activity of *Arabidopsis* Inward-Rectifying K^+^ Channel 1 (KAT1). GPA1 also interacts with carbonic anhydrases CA1 and CA4 to facilitate stomatal closure under high CO_2_ conditions (Zhang et al., 2025). Despite these advances, whether G proteins and their interaction with ethylene signaling participate in SL-mediated stomatal movement remains to be elucidated.

Hydrogen sulfide (H_2_S) and reactive oxygen species (ROS), especially H_2_O_2_, are well-established signaling molecules that participate in a wide range of plant physiological processes (Li et al., 2007; Jin et al., 2013; Li and He, 2015). Their interplay is particularly important under abiotic stresses such as drought and osmotic stress. For example, under drought conditions, phospholipase Dα1 (PLDα1) upregulates the expression of the H_2_S-synthesizing enzyme LCD, thereby promoting H_2_S production, which contributes to drought-induced stomatal closure—a process associated with H_2_O_2_ accumulation in guard cells (Wang et al., 2024). Both H_2_O_2_ and H_2_S act as key signaling intermediates in stomatal movement triggered by diverse factors, including ABA (Pei et al., 2000), MeJA (Suhita et al., 2004), BR (Shi et al., 2015; Ma et al., 2021, 2022), SL (Lv et al., 2018; Ma et al., 2024), ethylene (Hou et al., 2013), darkness (She et al., 2004; Ma et al., 2018), salt stress (Ma et al., 2019b), and cadmium chloride (CdCl_2_; Ma et al., 2019a). In *Arabidopsis*, BR-induced stomatal closure depends on AtrbohF-derived H_2_O_2_ generation, followed by AtL-CDes-/AtD-CDes-catalyzed H_2_S production (Ma et al., 2021). Similarly, SL-induced stomatal closure requires NADPH oxidase-dependent H_2_O_2_ synthesis and subsequent L-/D-CDes-derived H_2_S production (Ma et al., 2024). Notably, H_2_S can trigger H_2_O_2_ accumulation in guard cells independently of the classical NADPH oxidase RBOHD, indicating that H_2_S possesses an autonomous capacity to regulate ROS production in stomatal immunity (Scuffi et al., 2025). Despite these advances, the specific functions of H_2_O_2_ and H_2_S, as well as their relationship with ethylene and G proteins in SL-triggered stomatal closure, remain to be fully elucidated.

While SL are known to induce stomatal closure in *A. thaliana*, the signaling mechanism involving ethylene, Gα protein, H_2_O_2_, and H_2_S remains unclear. Our findings demonstrate that these components collectively mediate SL-induced stomatal closure and propose the following signaling pathway: SL first promote ethylene biosynthesis, which activates Gα protein, subsequently triggering H_2_O_2_ production and H_2_S generation, finally leading to stomatal closure. Our findings provide a theoretical basis for further elucidating the signal transduction mechanisms underlying SL-regulated stomatal movement in plants, and also provide evidence for the involvement of SL in regulating plant responses to abiotic stress.

## Materials and methods

### Chemicals

Molecular probe 2’,7’-dichlorodihydrofluorescein diacetate (H_2_DCF-DA), (3aR*, 8bS*, E)-3-(((R*)-4-methyl-5-oxo-2,5-dihydrofuran-2-yloxy)methylene)-3,3a,4,8b-tetrahydro-2H-indeno[1,2-b]furan- 2-one (GR24), 2-(*N*-morpholine) ethanesulfonic acid (MES), aminooxyacetic acid (AOA), aminoethoxyvinyl glycine (AVG), 1-methylcyclopropene (1-MCP), 1-aminocyclopropane-1- carboxylic acid (ACC), cholera toxin (CTX), pertussis toxin (PTX), ascorbic acid (ASA), catalase (CAT), diphenylene iodonium (DPI), dithiothreitol (DTT), *N,N*-dimethyl-*p*-phenylenediamine dihydrochloride, L-cysteine (L-Cys) and D-cysteine (D-Cys) were purchased from Sigma-Aldrich. Unless stated otherwise, all other chemicals were of the highest analytical grade purchased from various Chinese suppliers.

### Plant materials

Seeds were surface-sterilized and sown on sterilized vermiculite. Plates were stratified in darkness for 2–4 days at 4 °C. After growing four euphylla, seedlings were potted in a mixture of 1:1 vegetative soil and vermiculite in the controlled environment chamber with a humidity of 80%, 16 h light/8 h dark cycle, and day/night temperature cycle of 22 °C/18 °C with a photon flux density of 100 μmol·m^-2^·s^-1^ PAR generated by cool white fluorescent tubes (Philips, New York, NY, USA). Seeds of *A. thaliana* ecotype Columbia-0 (Col-0), *Atl-cdes*, *Atd-cdes*, *AtrbohD*, *AtrbohF* and *AtrbohD/F* mutants were obtained from the Nottingham *Arabidopsis* Stock Centre (NASC, Nottingham, UK). Seeds of *gpa1-1*, *gpa1-2*, *cGα1* and *wGα1* mutants (background Wassilewskija, Ws) were obtained from Prof. Zhonglin Shang (Hebei Normal University). Seeds of *etr1–1* and *etr1–3* mutants were obtained from Associate Prof. Changhua Zhu (Nanjing Agricultural University). Seeds of the wild type (Ws) and *eto1–1* mutant were obtained from Prof. Junmin He (Shaanxi Normal University). *cGα1* plants were grown in the presence of 70 nmol·L^-1^ dexamethasone, *wGα1* plants were grown in the presence of 30 nmol·L^-1^ dexamethasone. Fully expanded leaves of the wild type and mutants at 4-6-week-old were used for the following experiments. All treatments described below were at 22 ± 2 °C in light (100 μmol·m^-2^·s^-1^).

### Stomatal bioassays

Stomatal bioassays were performed as described by McAinsh et al. (1996) with minor modifications. Briefly, the freshly prepared epidermal strips were incubated in MES buffer (10 mmol·L^-1^ MES, 50 mmol·L^-1^ KCl, 0.1 mmol·L^-1^ CaCl_2_, pH 6.15) in light (100 μmol·m^-2^·s^-1^) at 22 ± 2 °C for 3 h in order to promote stomatal opening. Once the stomata were fully open, the epidermis of the wild type or mutants was carefully peeled from the abaxial surfaces of the youngest, fully expanded leaves of seedlings and cut into pieces of 5 mm × 5 mm, and were incubated in MES buffer alone or containing various compounds or inhibitors at 22 ± 2 °C in light (100 μmol·m^-2^·s^-1^), and then stomatal aperture were recorded with a light microscope and an eyepiece graticule previously calibrated with a stage micrometre. Experiments were always started at the same time each day in order to avoid any potential rhythmic effects on stomatal aperture. The data are represented as means ± SEs of three replicates (n=90), each with 30 stomata.

### Measurement of H_2_S emission

Measurement of H_2_S emission was performed as described by Sekiya et al. (1982) and Hou et al. (2013) with minor changes. The leaves of the wild type or mutants were cut and treated with MES buffer alone or containing various compounds or inhibitors at 22 ± 2 °C in light (100 μmol·m^-2^·s^-1^) for 3 h, and then were used to measure H_2_S content. Firstly, 0.1 g treated leaves were taken out and ground in the presence of 0.9 mL 20 mmol·L^–1^ Tris-HCl (pH 8.0) buffer. After grinding and centrifuging for 15 min, the supernatant and a trap with 3 mL of zinc acetate were put into a test tube, and sealed quickly with a parafilm. After H_2_S was absorbed for 30 min at 37 °C, 100 μL 20 mmol·L^-1^ N, N-dimethyl-phenylene diamine dihydrochloride dissolved in 7.2 mol·L^-1^ HCl and 100 μL 30 mmol·L^-1^ FeCl_3_ dissolved in 1.2 mol·L^-1^ HCl were added into the trap. Finally, the absorbance was measured at 670 nm, and a calibration curve was made with known concentrations of H_2_S solution. Each treatment was repeated three times, and the data presented are means ± SEs of 9 measurements.

### L-/D-cysteine desulfhydrase activity measurements

L-/D-cysteine desulfhydrase activity measurements were performed as described by Riemenschneider et al. (2005). The leaves of the wild type or mutants were cut and treated with MES buffer alone or containing various compounds or inhibitors at 22 ± 2 °C in light (100 μmol·m^-2^·s^-1^) for 3 h, and then were used to measure L-/D-Cdes activity. The content of each component in the total volume of 1 mL includes: 100 µL 0.8 mmol·L^-1^ L-/D-cysteine, 400 µL 2.5 mmol·L^-1^ DTT, 400 µL 100 mmol·L^-1^ Tris-HCl, and 100 µL supernatant. Then 100 µL 20 mmol·L^-1^ N, N-dimethyl-p-phenylenediamine dihydrochloride dissolved in 7.2 mol·L^-1^ HCl and 100 µL 30 mmol·L^-1^ FeCl_3_ dissolved in 1.2 mol·L^-1^ HCl were added into the trap after reacting for 30 min at 37 °C. The rate of H_2_S released was indicated by the determination of absorbance at 670 nm. Moreover, the activities of L-/D-CDes were determined by the same method, but the pH of Tris-HCl buffer used by the former and latter was 9 and 8, respectively. Each treatment was repeated at least three times, and the data presented are means ± s.e. of 9 measurements.

### Treatment with 1-methylcyclopropene

For treatment with 1-MCP, 4.8 mg of 1-MCP was dissolved in 73 µL of distilled water in a tube, and the 4-6-week plants were laid with the tube in a closed chamber for 12 h. The final concentration of 1-MCP in the gas phase was expected to be 500 pl·L^-1^. After treatment, the rosette leaves were incubated in MES buffer under light, to open the stomata, before being treated as described above and used for subsequent experiments.

### RNA isolation and transcript analysis by RT-PCR

Total RNA was isolated from the treated leaves using Plant RNA Kit (Transgen, China) according to the manufacturer’s instructions. First-strand cDNA was synthesized with the One-Step gDNA Removal and cDNA Synthesis SuperMix (Transgen, China). The cDNA was then amplified by PCR with gene-specific primers for *ACS2*, *ACS4-9*, *ACS11*, *GPA1* and *ACTIN* at an annealing temperature of 55 °C. Primers used for RT-PCR with cycles are as follows: *ACTIN* (5’-CAAGGCCGAGTATGATG AGG-3’ and 5’-GAAACGCAGACGTAAGTAAAAAC-3’), *ACS2* (5’-AAAGCCCAAGAGTCCAATA A-3’ and 5’-AGACGAAACAAGTCCGAAAC-3’), *ACS4* (5’-CGACAACAACCCAAACCGAACT-3’ and 5’-TGGCACGACGAACCAGGAG-3’), *ACS5* (5’-TGGCACGACGAACCAGGAG-3’ and 5’-T CATTTCGTCGTTGGAGT-3’), *ACS6* (5’-GCTGATGAGATTTATGCTGCTA-3’ and 5’-CACGCCATA GTTCGGTTTCC-3’), *ACS7* (5’-AACCATTTCCAGATAACCCC-3’ and 5’-AACACTCAATCCCTGC CTTC-3’), *ACS8* (5’-GTCGGAAAATAGAGGAAATCG-3’ and 5’-CTGCGGAGACAACAAAATCAT -3’), *ACS9* (5’-GGGATGGGAAGAATACGA-3’ and 5’-ACATTGTGCCAAGAGGGT-3’), *ACS11* (5’-C TTTCTTATCCCTGCACCTTA-3’ and 5’-GAACAAACCTGCGTTACTCTT-3’), *GPA1* (5’-CACAGG CTGCTGAAATCG-3’ and 5’-CTCCCACAGGGCTGAACT-3’), Bands in gels from RT-PCR experiments were measured on images with the B_IO_D_OC_-IT imaging system.

### Quantitative real-time PCR analysis

Total RNA was isolated from the treated leaves using Plant RNA Kit (Transgen, China) according to the manufacturer’s instructions. First-strand cDNA was synthesized with the One-Step gDNA Removal and cDNA Synthesis SuperMix (Transgen, China). Quantitative real-time PCR was performed using the Green qPCR SuperMix (+Dye I/+Dye II) (Perfect Real Time; Transgen, China). PCR was performed using a CFX 96 Real-Time system (Bio-Rad, Hercules, CA, United States) with the following standard cycling conditions: 94 °C for 30 s, 94 °C for 5 s, 60 °C for 30 s for 40 cycles. The primer sequences used in the study are as follows: *AtL-CDes* (5’-TGTA TGTGAGGAGGAGGC-3’ and 5’-GTTTCATACTGATGCTGCTC-3’), *AtD-CDes* (5’-CATGCCATGGCAATGAGAGGACGAAGCT TGACACTCTC-3’ and 5’-CGGGATCCCTAGAACATTTTCCCAACACCATCTT-3’) and *ACTIN* (5’-C AAGGCCGAGTATGATGAGG-3’ and 5’-GAAACGCAGACGTAAGTAAAAAC-3’). We used a cycle threshold 2^(−ΔΔC(T))^-based method for the relative quantification of gene expression.

### Ethylene measurement

After the aforementioned treatments, the rosette leaves were enclosed in a vial containing filter paper moistened by MES buffer with the corresponding compounds or inhibitors, under light conditions. After treatment for 3 h, the gas in the vial was gathered with a syringe, and then the concentration of ethylene was measured with an Agilent 6890 N gas chromatograph system, equipped with a flame ionization detector on an HP-5 capillary column. In each treatment, six leaves from different plants were measured, and the treatment was repeated at least three times. Data are presented as means ± SEs (n = 9).

### Measurement of endogenous H_2_O_2_ levels

H_2_O_2_ levels were monitored by using the fluorescent indicator dye 2′,7′-dichlorodihydrofluorescein diacetate (H_2_DCF-DA), as previously described by Allan and Fluhr (1997) with minor modifications. After the aforementioned treatments, the epidermal strips were immediately loaded with 50 μmol·L^-1^ H_2_DCF-DA in Tris-KCl loading buffer (10 mmol·L^-1^ Tris, 50 mmol·L^-1^ KCl, pH 7.2) for 10 min in darkness at 25 °C, then the excessive dye was washed off with fresh Tris-KCl loading buffer, and the epidermal strips were immediately examined by luminescence microscope (Olympus BX53, U-RFLT50, JAPAN) with the following settings: 450–490 nm of excitation, 520–560 nm of emission. Images thus acquired were analysed with Leica image software and processed with Photoshop 7.0 (Adobe, San Jose, CA, USA). The selected images here represented the same results from all three time measurements.

### Statistical analysis

The statistical significance of treatments was checked using one-way ANOVA followed by Duncan’s multiple range test. The data were considered statistically significant when *P*-values were below 0.05.

## Results

### Ethylene mediates SL-induced stomatal closure

**Figure 1:**
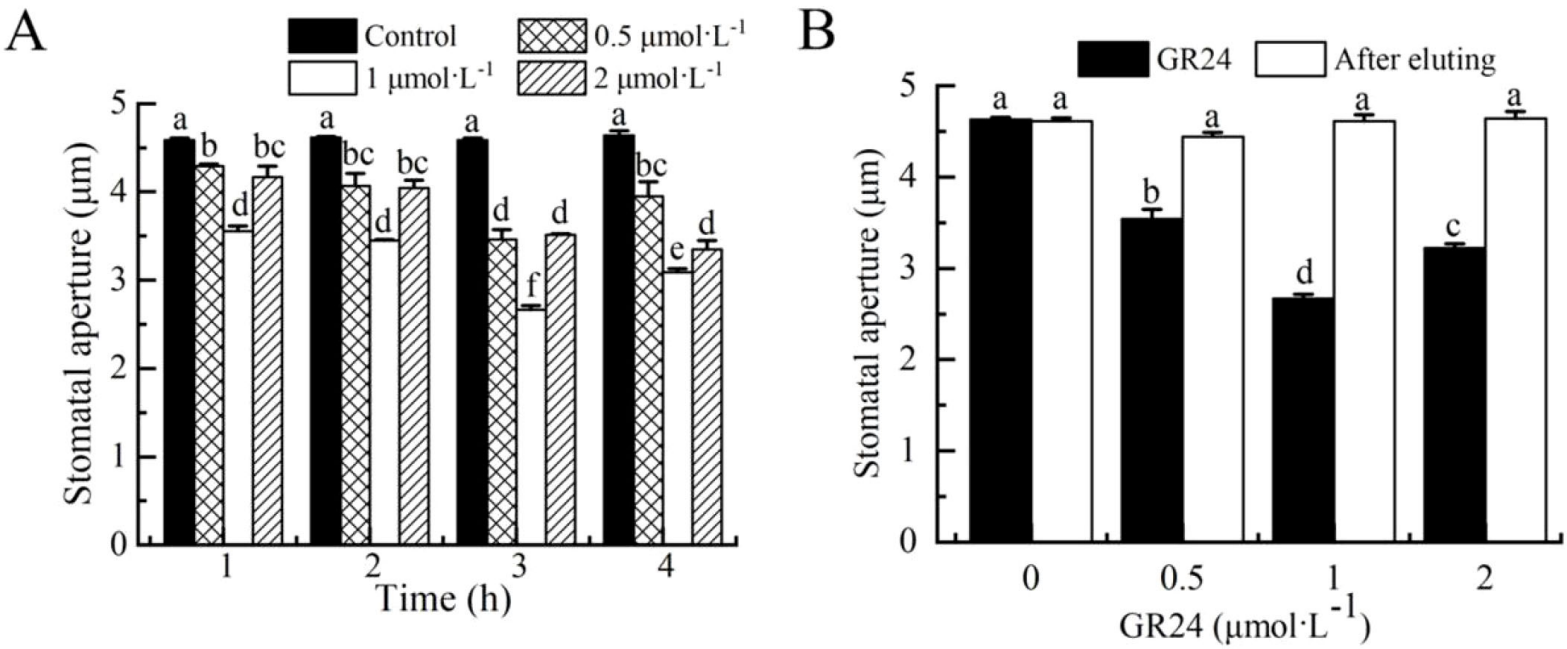
Effects of SL on stomatal aperture in *Arabidopsis* wild-type (WT) Col-0. **(A)** Epidermal strips were incubated in MES buffer without or with GR24 for indicated times. **(B)** Epidermal strips incubated with 1 µmol·L^-1^ GR24 for 3 h were transferred to MES buffer alone for another 3h After treatment, stomatal apertures were recorded. The Data are represented as means ± SEs of three replicates (n = 90, 30 stomata each). Means with different letters are significantly different at *P* < 0.05.

To examine the ability of SL to close stomata, we tested a synthetic SL analog, GR24, at various concentrations and exposure times on stomatal aperture in the wild type (Col-0). GR24 significantly induced stomatal closure in a concentration- and time-dependent manner within 3 h of treatment. Treatment with 1 µmol·L^-1^ GR24 for 3 h reduced 42% stomatal aperture compared with the control, which was identified as the most effective concentration and duration (**Figure 1A**). Stomatal responses to GR24 were reversible upon elution treatment (**Figure 1B**). The results here clearly showed that SL could effectively close stomata in *A. thaliana*.

**Figure 2:**
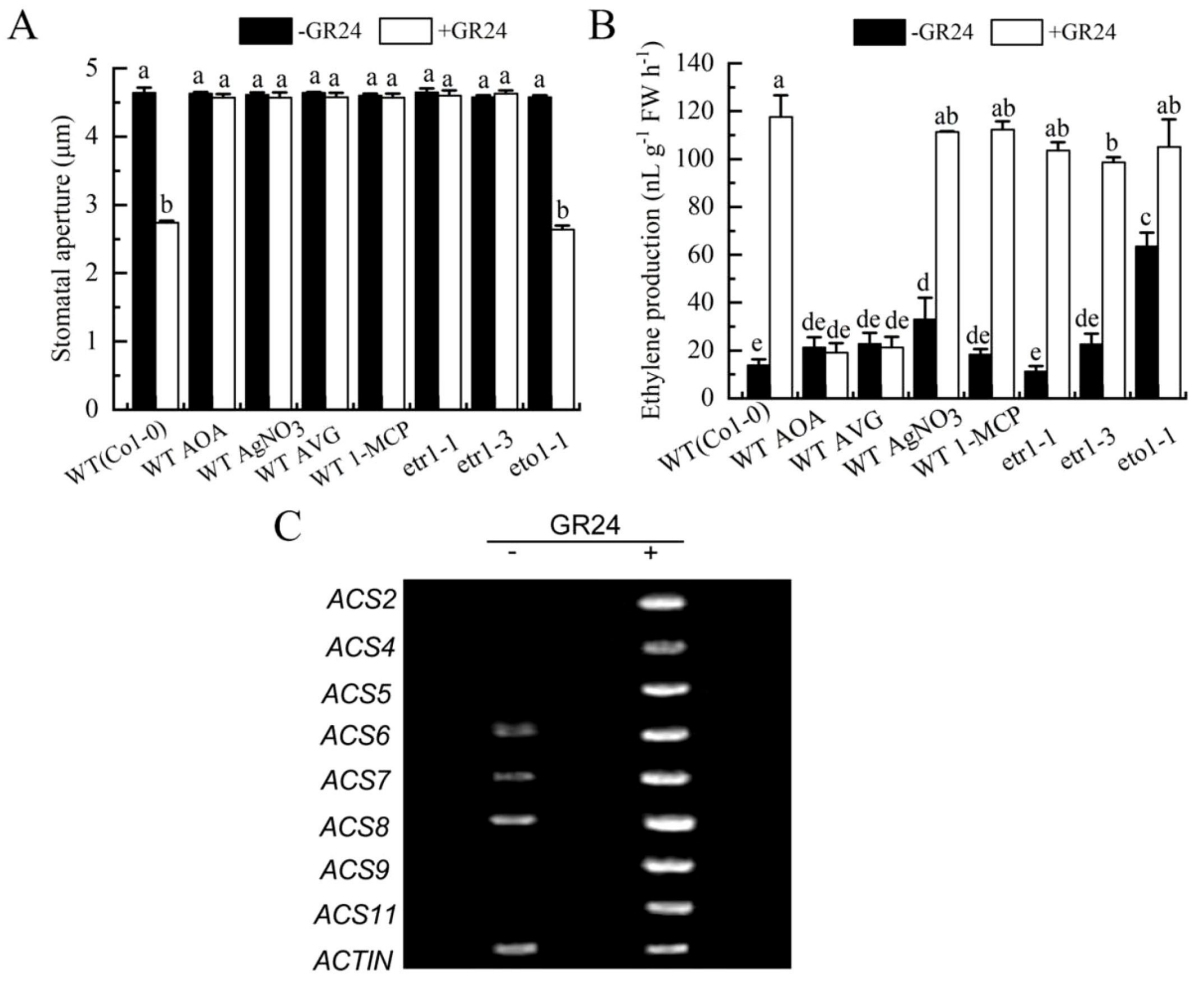
Ethylene mediates SL-induced stomatal closure. *Arabidopsis* WT (Col-0) were pretreated without or with 500 pl·L^-1^ 1-MCP in a closed chamber, or not, and leaves were incubated in MES buffer alone, or with 50 µmol·L^-1^ AOA, 50 µmol·L^-1^AVG, 10 µmol·L^-1^ AgNO_3_, without or with GR24 for 3 h, and leaves of mutants *etr1-1*, *etr1–3* and *eto1–1* were incubated in MES buffer without or with GR24 for 3h **(A)** Stomatal apertures were measured. **(B)** The treated leaves were enclosed in a vial for another 3 h, and ethylene was collected and quantified. The Data in A and B are represented as means ± SEs of three replicates (n = 90 for A; n = 3 for B). Means with different letters are significantly different at *P* < 0.05. **(C)** Wild-type leaves were treated with MES buffer alone or containing GR24 for 3 h, and transcript levels of *ACS2* and *ACS4–11* were analyzed by RT-PCR.

To elucidate the role of ethylene in SL-triggered stomatal closure, we analyzed the effect of GR24 on stomatal aperture in the wild type, ethylene-overproducing mutant *eto1-1*and ethylene-insensitive mutants *etr1–1* and *etr1-3*. GR24 induced stomatal closure in the wild type and *eto1–1* mutant, whereas this response was completely abolished in both *etr1–1* and *etr1–3* mutants, which harbor distinct mutations (Cys65Tyr and Ala31Val, respectively) in the ethylene receptor ETR1 (Chang et al., 1993; **Figure 2A**). Moreover, GR24-induced stomatal closure was inhibited by ethylene biosynthesis inhibitors (AOA and AVG) and ethylene perception inhibitors (AgNO_3_ and 1-MCP) (**Figure 2A**). These results clearly demonstrated that ethylene might be an essential signaling component in SL-triggered stomatal closure.

To further clarify the role of ethylene in SL-triggered stomatal closure, we measured ethylene production and ACC synthase (ACS) gene expression in wild-type leaves. GR24 treatment markedly enhanced ethylene production in the wild type, and this enhancement was substantially suppressed by AOA and AVG, but unaffected by silver and 1-MCP (**Figure 2B**). Similarly, GR24 promoted ethylene production in *etr1-1*, *etr1-3*, as well as in *eto1–1* mutants, to an extent comparable to that in the wild type (**Figure 2B**). Additionally, GR24 treatment significantly upregulated the expression of *ACS2*, *ACS4–9* and *ACS11* in the wild type (**Figure 2C**). These results indicated that ethylene played a vital role in SL-triggered stomatal closure in *A. thaliana*. Notably, AOA, AVG, AgNO_3_, 1-MCP, and the ETR1 mutation influenced GR24-induced stomatal closure and ethylene synthesis align with their known actions on ethylene biosynthesis or signaling pathways.

### Heterotrimeric G protein α-subunit is involved in SL-induced stomatal closure

**Figure 3:**
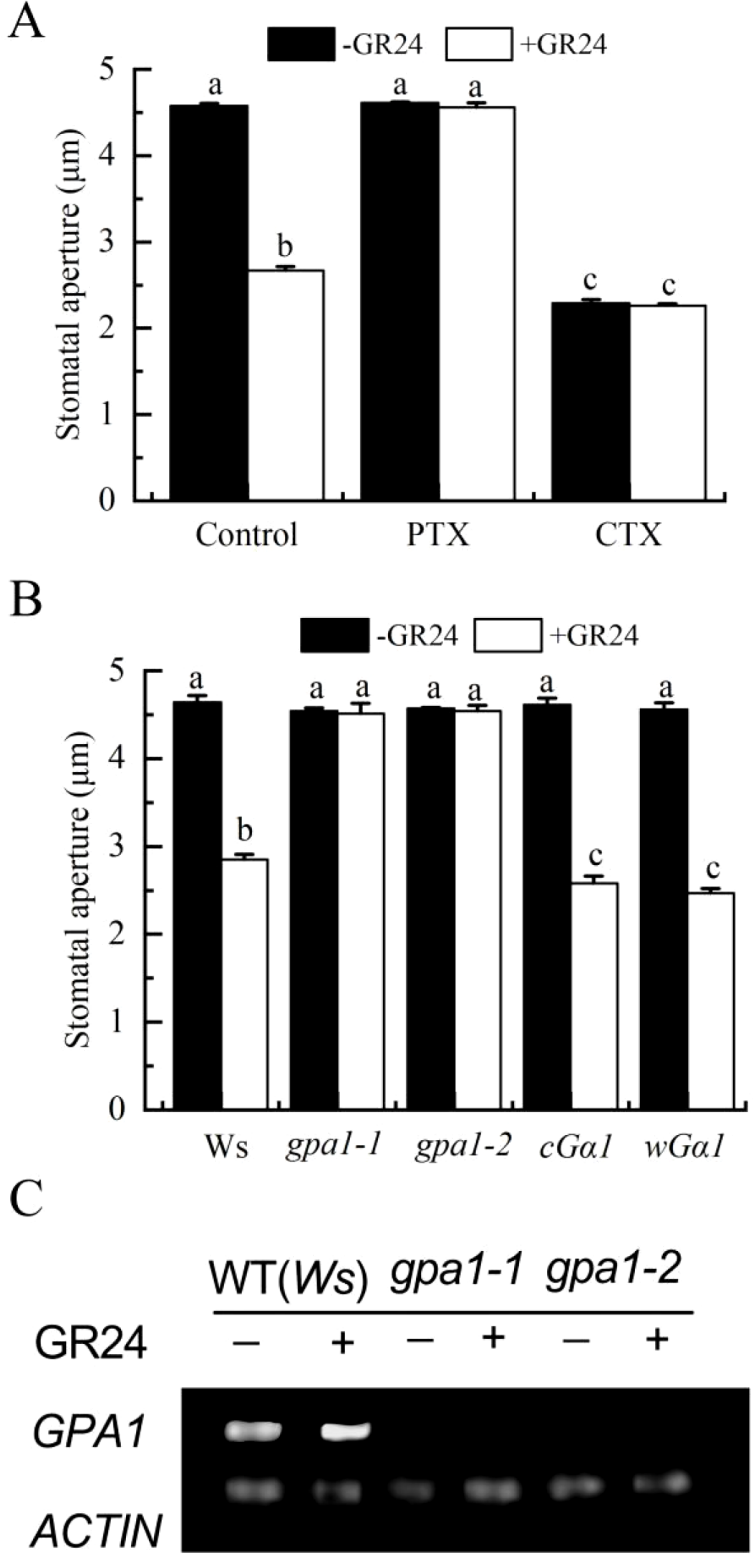
Gα is crucial for SL-induced stomatal closure. **(A)** Epidermal strips of WT (Ws) were incubated in MES buffer alone or in presence of 400 ng·ml^-1^ PTX, or 400 ng·ml^-1^CTX, without or with GR24 for 3h **(B)** Epidermal strips of WT (Ws) and mutants *gpa1-1*, *gpa1-2*, *cGα1*, and *wGα1* were incubated without or with GR24 for 3h After treatment, stomatal apertures were recorded. See **Figure 1** for further details in **(A–C)**. Leaves of WT (Ws) and mutants *gpa1–1* and *gpa1–2* were treated with MES buffer alone or containing GR24 for 3 h, *GPA1* transcript levels were analyzed by RT-PCR. Each assay was repeated at least three times.

To investigate the potential involvement of G proteins in SL-induced stomatal closure, we examined the effects of Gα inhibitor (pertussis toxin, PTX) and Gα activator (cholera toxin, CTX) on stomatal aperture in the wild-type ecotype Wassilewskija (Ws). GR24-induced stomatal closure was significantly suppressed by PTX. Conversely, CTX induced stomatal closure, mirroring the effect of GR24 (**Figure 3A**). These results indicated that Gα signaling might contribute to SL-induced stomatal closure in *A. thaliana*.

To further validate the involvement of Gα in SL-induced stomatal closure, stomatal responses to GR24 were assessed in the *Arabidopsis* Gα mutants *gpa1–1* and *gpa1-2* (Ullah et al., 2001), the Gα-overexpressing lines cGα and wGα1, and the corresponding wild type. In **Figure 3B**, GR24-induced stomatal closure was completely abolished in *gpa1–1* and *gpa1–2* mutants but remained unaffected in *cGα1* and *wGα1* mutants compared to the wild type. Furthermore, GR24 treatment upregulated GPA1 transcript levels in wild-type leaves but not in the *gpa1* mutants (**Figure 3C**). Together with the pharmacological evidence presented in **Figures 3A**, these results demonstrated that Gα mediated SL-triggered stomatal closure in *Arabidopsis*.

### Ethylene promotes both H_2_O_2_ and H_2_S synthesis in stomatal closure induced by SL

**Figure 4:**
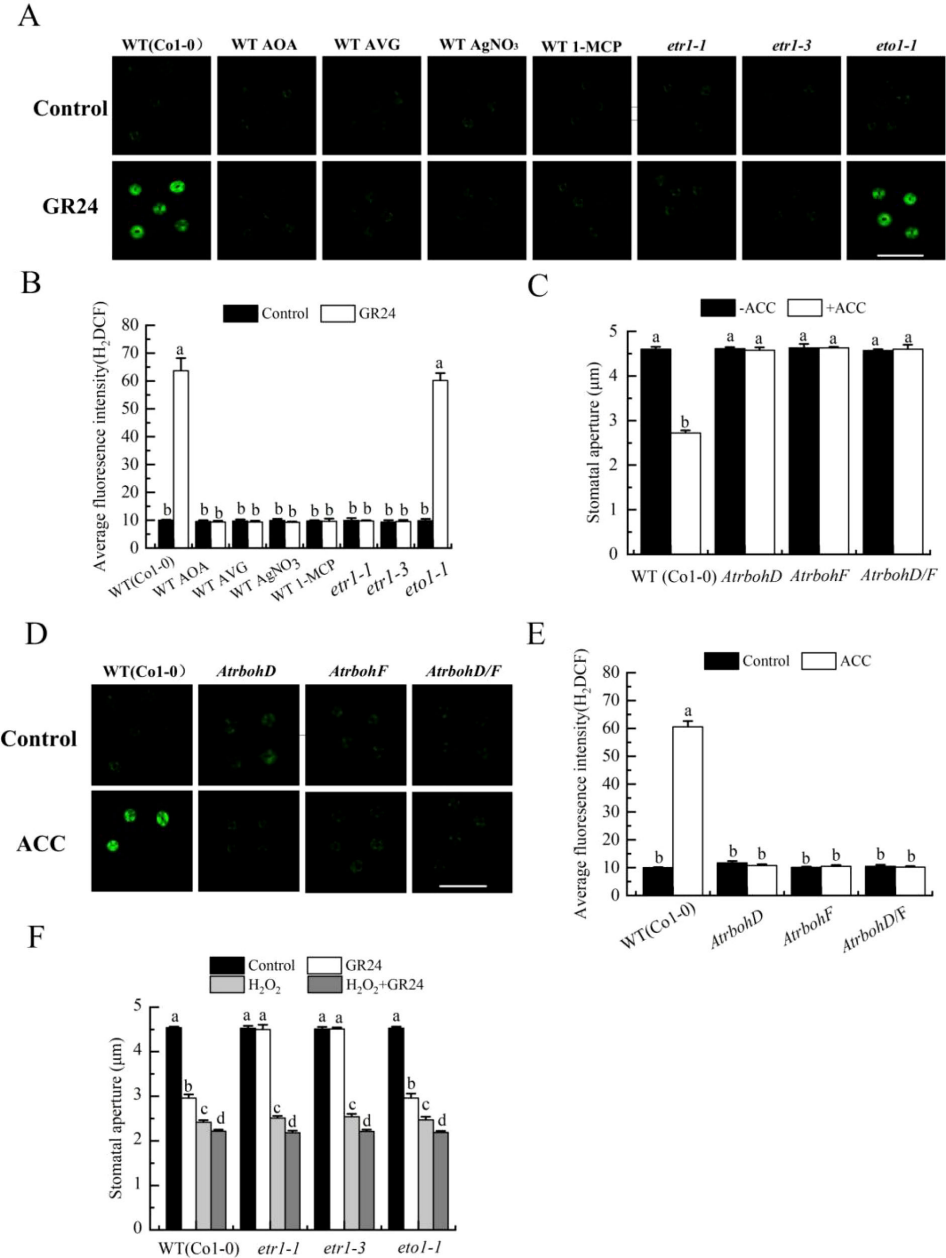
Ethylene mediates SL-induced stomatal closure via inducing H_2_O_2_ production. **(A, B)***Arabidopsis* WT (Col-0) were pretreated without or with 500 pl·L^-1^ 1-MCP in a closed chamber, or not, and epidermal strips of WT (Col-0) were incubated in MES buffer alone (Control) or in the presence of AOA, AVG, or AgNO_3_ (same concentrations as in **Figure 2**), without or with GR24 for 3 h; epidermal strips of mutants *etr1-1*, *etr1–3* and *eto1–1* were treateded in MES buffer alone without or with GR24 for 3h Fluorescence images **(A)** and H_2_DCF pixel intensities in guard cells **(B)** were recorded after 10 min darkness loading with 50 µmol·L^-1^ H_2_DCF-DA. **(C)** Epidermal strips of WT (Col-0) and mutants *AtrbohD*, *AtrbohF* and *AtrbohD/F* were incubated in MES buffer alone or containing 100 µmol·L^-1^ ACC for 3h **(D, E)** Epidermal strips of the same genotypes with C were treated with MES buffer alone or containing ACC for 3h Fluorescence images **(D)** and H_2_DCF pixel intensities in guard cells **(E)** were recorded after H_2_DCF-DA loading. **(F)** Epidermal strips of WT (Col-0) and mutants *etr1-1*, *etr1–3* and *eto1–1* were incubated in MES buffer alone (Control) or with GR24, 100 µmol·L^-1^ H_2_O_2_, or both for 3h After treatment, stomatal apertures were recorded **(C, F)**. Scale bars in A and D: 40 µm. The Data in **(B, C, E, F)** are represented as means ± SEs of three replicates [n = 90 for **(C, F)**; n = 3 for **(B, E)**]. Means with different letters are significantly different at *P* < 0.05 in **(B, C, E, F)**.

Previous studies showed that H_2_O_2_ synthesis mediates SL-induced stomatal closure (Lv et al., 2018; Ma et al., 2024). To further explore the relationship between ethylene and H_2_O_2_ in this process, we first examined the role of ethylene in H_2_O_2_ production of guard cells during SL signaling. AOA, AVG, AgNO_3_ and 1-MCP obviously prohibited GR24-induced H_2_O_2_ accumulation in the wild type (Col-0) (**Figures 4A, B**). Furthermore, GR24 triggered H_2_O_2_ production in the wild type and *eto1–1* mutant, but failed to do so in *etr1-1*and *etr1–3* mutants (**Figures 4A, B**). We next assessed whether ethylene acts through H_2_O_2_ by testing the response of *AtrbohD*, *AtrbohF* and *AtrbohD/F* mutants to the ethylene precursor ACC. ACC-induced stomatal closure and H_2_O_2_ accumulation observed in the wild type were completely abolished in these NADPH oxidase mutants (**Figures 4C–E**). Finally, to further determine whether H_2_O_2_ functions downstream of ethylene, we applied exogenous H_2_O_2_ to *etr1–1* and *etr1–3* mutants treated with GR24 (Wang et al., 2004). While GR24 alone did not close stomata in these *etr1–1* and *etr1–3* mutants, exogenous H_2_O_2_ fully restored stomatal closure (**Figure 4F**). Collectively, these results demonstrated that ethylene acted upstream of H_2_O_2_ production in SL-induced stomatal closure in *Arabidopsis*.

**Figure 5:**
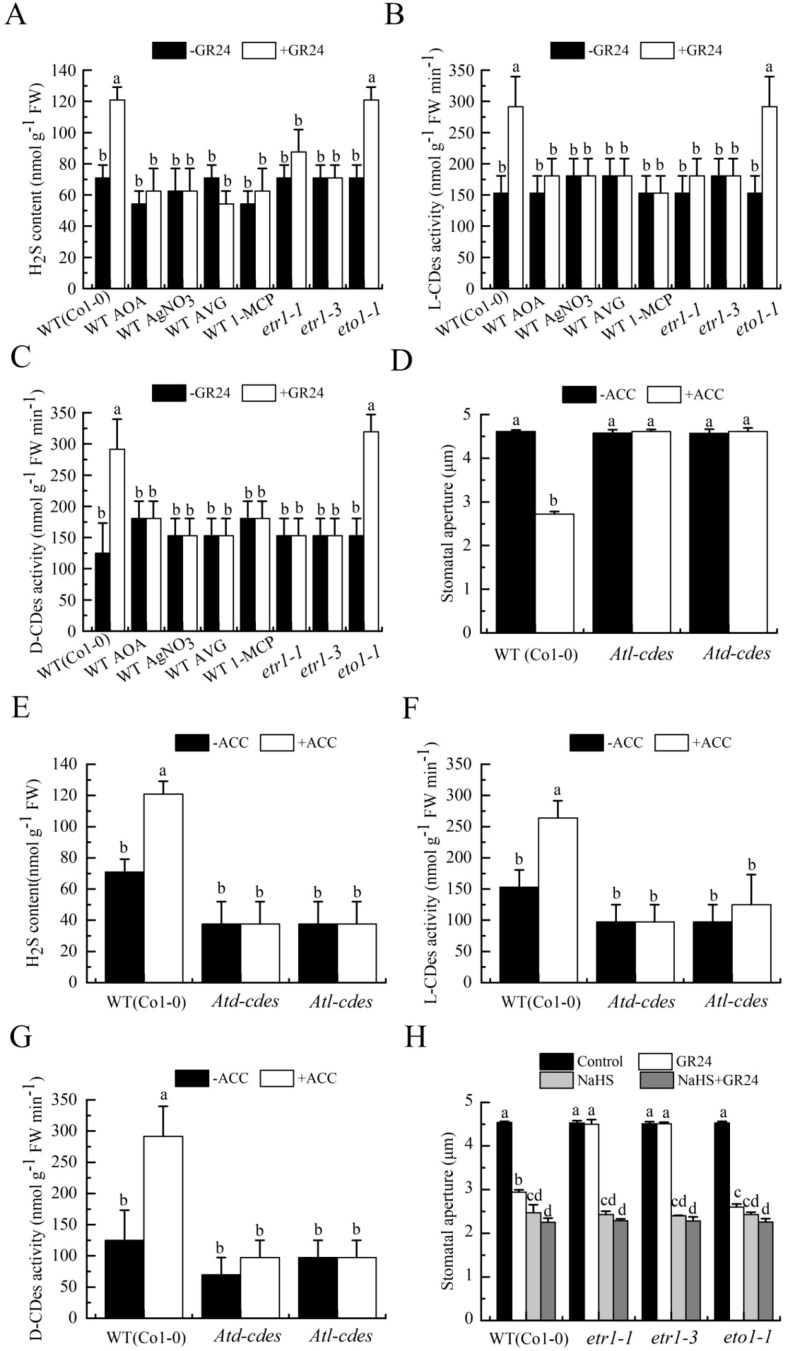
Ethylene mediates SL-induced stomatal closure via inducing H_2_S synthesis. **(A–C)**. *Arabidopsis* WT (Col-0) were pretreated without or with 500 pl·L^-1^ 1-MCP in a closed chamber, or not, and leaves of WT (Col-0) treated in MES buffer or containing GR24 in the presence of AOA, AVG, AgNO_3_, or 1-MCP (same concentrations as in **Figure 2**.), and leaves of mutants *etr1-1*, *etr1-3*, and *eto1–1* in MES buffer without or with GR24 for 3 h. **(D)**. Epidermal strips of WT (Col-0) and mutants *Atl-cdes* and *Atd-cdes* were incubated in MES buffer or containing ACC for 3 h. **(E–G)**. Leaves of WT (Col-0) and mutants *Atl-cdes* and *Atd-cdes* were treated in MES buffer without or with ACC for 3 h. **(H)**. Epidermal strips of WT (Col-0) and mutants *etr1-1*, *etr1-3*, and *eto1–1* were incubated in MES buffer alone (Control), or containing GR24, 100 µmol·L^-1^ NaHS, or both for 3 h. After treatment, stomatal apertures were recorded **(D, H)**; and samples (0.1 g) were taken for H_2_S content **(A, E)**, L-CDes activity **(B, F)** and D-CDes activity **(C, G)**. The Data in **(A–H)** are represented as means ± SEs of three replicates [n = 9 for **(A–C, E–G)**; n = 90 for **(D, H)**]. Means with different letters are significantly different at *P* < 0.05.

H_2_S has been demonstrated to participate in SL-induced stomatal closure in *Arabidopsis* (Ma et al., 2024). To further study whether ethylene is related to H_2_S synthesis during this process, we first examined the effects of GR24 on H_2_S generation and the activities of L- and D-cysteine desulfhydrases (L-/D-CDes) in leaves. Using wild-type (Col-0) plants treated with or without AOA, AVG, AgNO_3_, and 1-MCP, or mutants *etr1-1*, *etr1–3* and *eto1-1*, we observed that GR24 significantly increased H_2_S content and L-/D-CDes activity in both Col-0 and *eto1-1*. These increases were substantially suppressed in Col-0 by AOA, AVG, AgNO_3_, and 1-MCP, while GR24 had no effect on *etr1–1* and *etr1–3* mutants (**Figures 5A–C**).

We further tested the impact of ACC on stomatal aperture, H_2_S content, and L-/D-CDes activity in *Atl-cdes* and *Atd-cdes* mutants. ACC induced stomatal closure, enhanced H_2_S accumulation, and elevated L-/D-CDes activity in the wild type, but these responses were abolished in both *Atl-cdes* and *Atd-cdes* mutants (**Figures 5D–G**). Together, these results indicated that ethylene participated in SL-triggered stomatal closure through promoting H_2_S synthesis. Additionally, GR24 failed to close stomata in *etr1–1* and *etr1–3* mutants, whereas application of NaHS restored GR24-induced stomatal closure in these genotypes (**Figure 5H**). This confirmed that H_2_S acted downstream of ethylene to mediate SL-induced stomatal closure in *Arabidopsis*, with L-CDes and D-CDes catalyzing H_2_S production in this process.

### Gα activates H_2_O_2_ production and H_2_S synthesis in SL-induced stomatal closure

**Figure 6:**
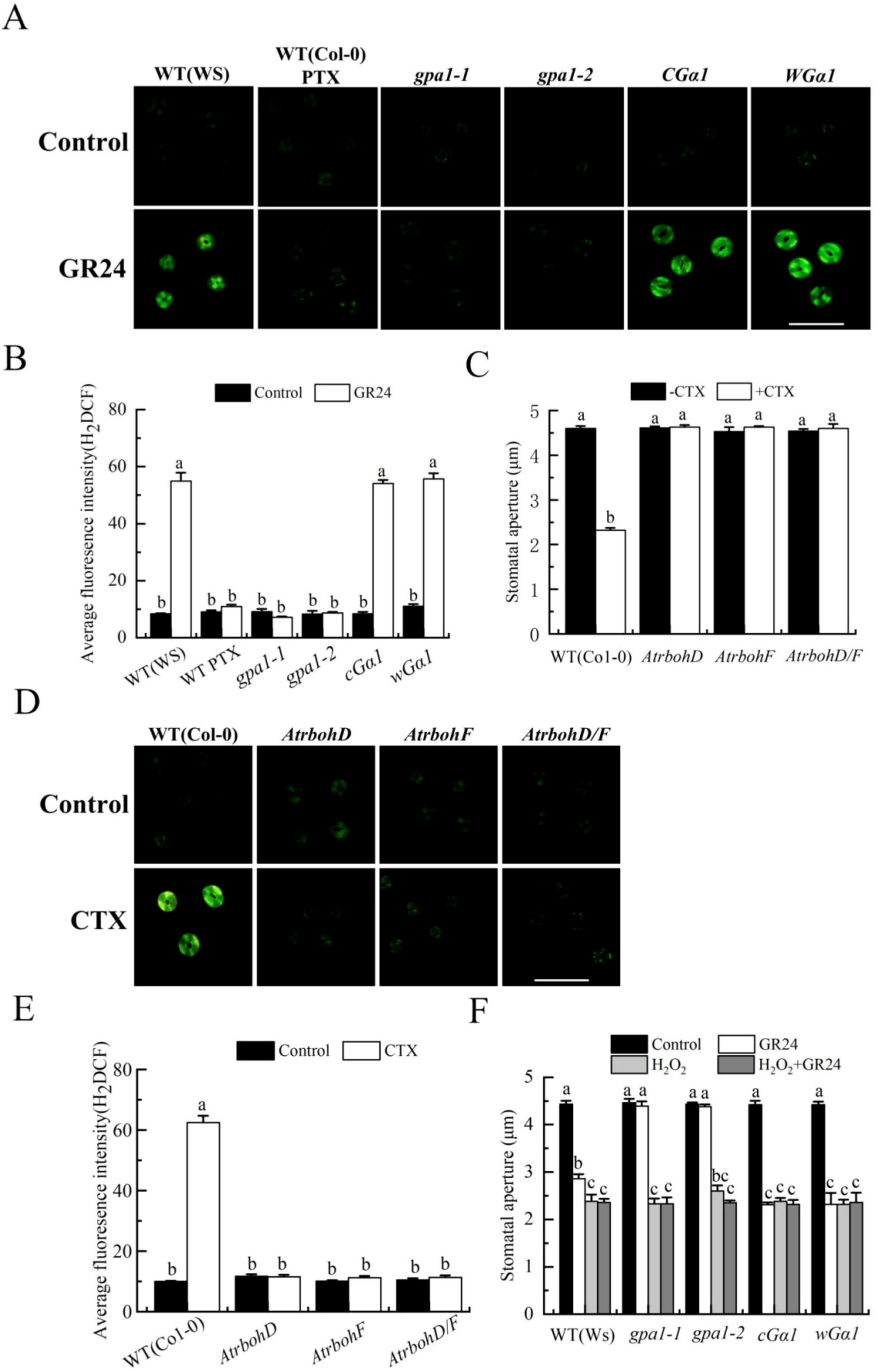
GPA1 mediates SL-induced stomatal closure through activating H_2_O_2_ production. **(A, B)** Epidermal strips of WT (Ws) were incubated in MES buffer alone, or containing PTX without or with GR24, and epidermal strips of mutants *gpa1-1*, *gpa1-2*, *cGa1* and *wGa1* were incubated in MES buffer alone, or containing GR24 for 3 h Fluorescence images **(A)** and H_2_DCF-DA pixel intensities in guard cells **(B)** were recorded after H_2_DCF-DA loading. C-E. Epidermal strips of WT (Col-0) and mutants *AtrbohD*, *AtrbohF* and *AtrbohD/F* were incubated in MES buffer alone, or containing CTX for 3 h After treatment, stomatal apertures were measured **(C)**, fluorescence images **(D)** and H_2_DCF pixel intensities in guard cells **(E)** were recorded after H_2_DCF-DA loading. **(F)** Epidermal strips of WT (Ws) and mutants *gpa1-1*, *gpa1-2*, *cGα1* and w*Gα1* were incubated in MES buffer alone (Control) or with GR24, H_2_O_2_, or both for 3h After treatment, stomatal apertures were recorded **(C, F)**. Scale bars in **(A, D)**: 40 µm. See **Figure 4** for further details in **(B, C, E, F)**.

Having established the involvement of Gα in SL-induced stomatal closure in *Arabidopsis*, we next investigated its relationship with H_2_O_2_ in this process. First, we examined the effect of GR24 on H_2_O_2_ production in guard cells of the wild type (Ws) either untreated or treated with PTX, as well as in mutants *gpa1-1*, *gpa1-2*, *cGα1* and *wGα1*. GR24 elevated H_2_O_2_ levels in guard cells of the wild type, *cGα1*, and *wGα1* mutants, but this increase was markedly inhibited by PTX in the wild type. In contrast, GR24 failed to induce H_2_O_2_ production in *gpa1–1* and *gpa1–2* mutants (**Figures 6A, B**). We further assessed the impact of CTX on stomatal aperture and H_2_O_2_ synthesis in guard cells of *AtrbohD*, *AtrbohF* and *AtrbohD/F* mutants. CTX triggered stomatal closure and H_2_O_2_ accumulation in wild-type (Col-0) guard cells, whereas these responses were fully abolished in all three *Atrboh* mutants (**Figures 6C–E**). Finally, we tested whether exogenous H_2_O_2_ could restore GR24-induced stomatal closure in Gα-impaired mutants. While GR24 couldn’t close stomata in *gpa1–1* and *gpa1–2* mutants, application of H_2_O_2_ largely restored stomatal closure in these lines under GR24 treatment (**Figure 6F**). Together, these results indicated that Gα acted upstream of H_2_O_2_ production in SL-triggered stomatal closure in *Arabidopsis*.

**Figure 7:**
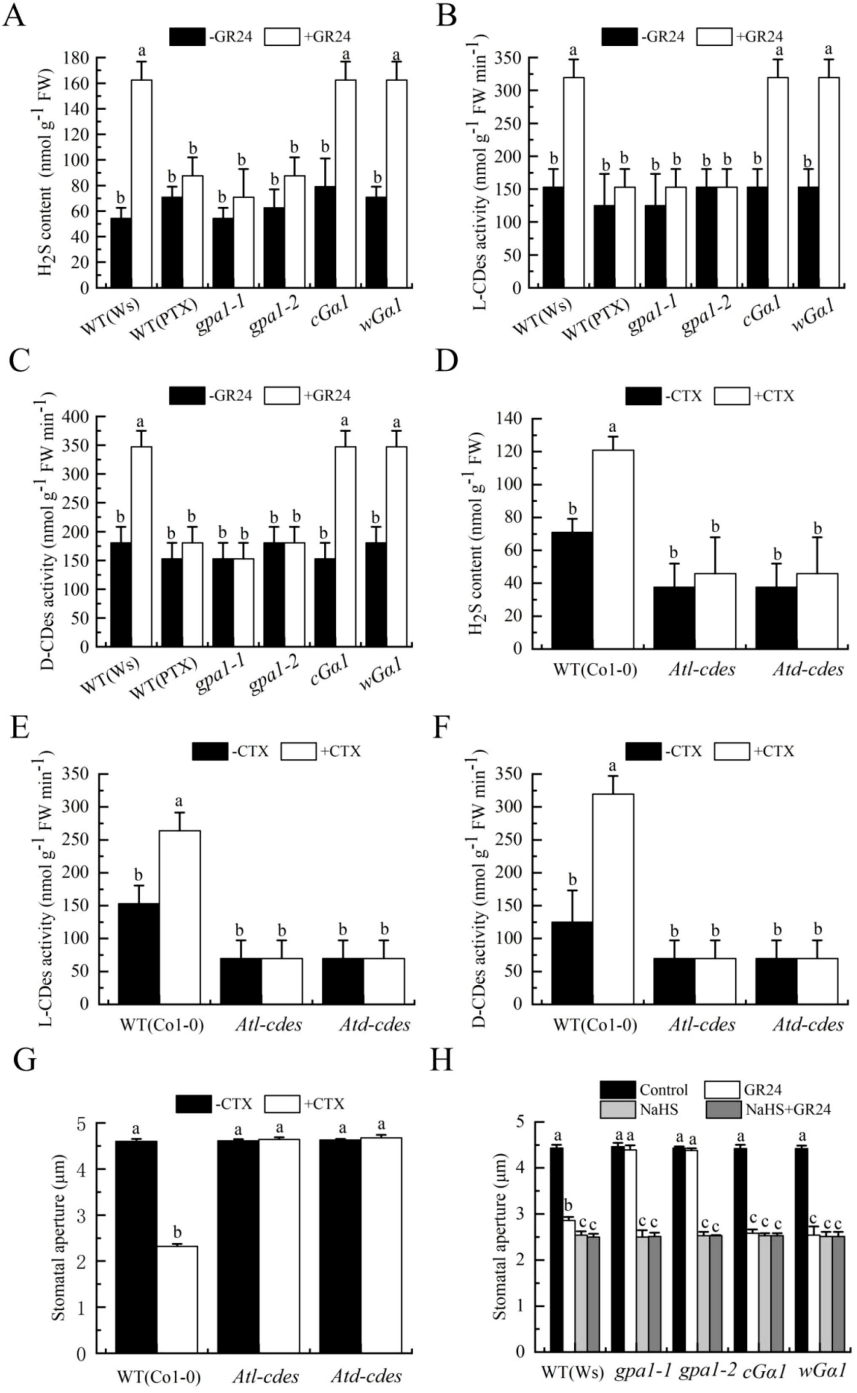
GPA1 mediates SL-induced stomatal closure via activating H_2_S synthesis. **(A–C)** Leaves WT (Ws) were treated in MES buffer alone, or containing PTX without or with GR24 for 3 h, and leaves of mutants *gpa1-1*, *gpa1-2*, *cGα1* and w*Gα1* were treated in MES buffer alone, or containing GR24 for 3 h. **(D–F)** Leaves of WT (Col-0) and mutants *Atl-cdes* and *Atd-cdes* were treated in MES buffer alone, or containing CTX for 3 h. **(G)** Epidermal strips of WT (Col-0) and mutants *Atl-cdes* and *Atd-cdes* were incubated in MES buffer alone, or containing CTX for 3 h. **(H)** Epidermal strips of WT (Ws) and mutants *gpa1-1*, *gpa1-2*, *cGα1* and w*Gα1* were incubated in MES buffer alone, or containing GR24, NaHS, or both for 3 h. After treatment, samples (0.1 g) were taken for H_2_S content **(A, D)**, L-CDes activity **(B, E)** and D-CDes activity **(C, F)**, and stomatal apertures were recorded **(G, H)**. The Data in **(A–H)** are represented as means ± SEs of three replicates [n = 9 for **(A–F)**; n = 90 for **(G, H)**]. Means with different letters are significantly different at *P* < 0.05.

Given the established roles of Gα and H_2_S in strigolactone (SL)-induced stomatal closure, the interrelationship between Gα and H_2_S in the process through pharmacological genetic approaches was further probed. First, we examined the effects of GR24 on H_2_S production and L-/D-CDes activity in leaves of the wild type (Ws) in the presence or absence of PTX, as well as in Gα mutants (gpa1-1, gpa1-2) and Gα-overexpressing lines (cGα1, wGα1). GR24 significantly increased H_2_S accumulation and enhanced both L- and D-CDes activity in the wild type, cGα1, and wGα1, whereas these effects were blocked by PTX in the wild type and were absent in *gpa1–1* and *gpa1–2* mutants (**Figures 7A–C**). These results indicated that Gα activation was required for SL-induced H_2_S synthesis. We next tested whether CTX could induce H_2_S production and whether this depended on functional L- and D-CDes. CTX increased H_2_S content and L-/D-CDes activity in wild-type leaves, but these responses were markedly suppressed in *Atl-cdes* and *Atd-cdes* mutants (**Figures 7D–F**). To further confirm that H_2_S acted downstream of Gα, we examined stomatal responses in relevant mutants. CTX-induced stomatal closure was impaired in *Atl-cdes* and *Atd-cdes* mutants (**Figure 7G**). Conversely, exogenous H_2_S (NaHS) substantially restored GR24-triggered stomatal closure in *gpa1–1* and *gpa1–2* mutants, while having little additional effect in Gα-overexpressing lines (**Figure 7H**). Together, these findings demonstrated that Gα promoted H_2_S synthesis via L- and D-CDes during SL-induced stomatal closure in *Arabidopsis*.

### Gα is essential for the action of ethylene in SL-induced stomatal closure

**Figure 8:**
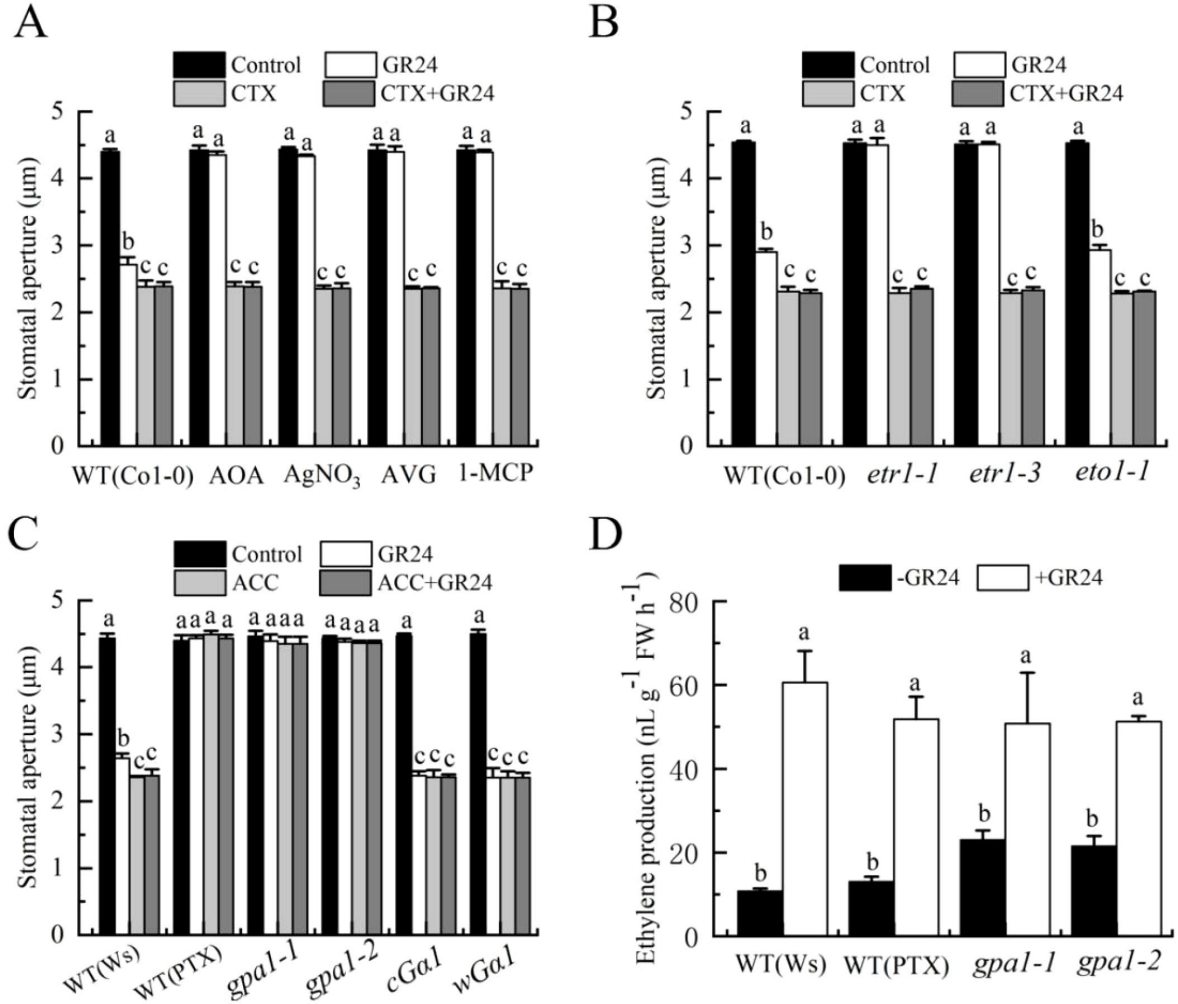
GPA1 mediates the action of ethylene in SL-induced stomatal closure. **(A)***Arabidopsis* WT (Col-0) were pretreated without or with 500 pl·L^-1^ 1-MCP in a closed chamber, or not, and epidermal strips of WT (Col-0) were incubated in MES buffer alone, or containing AOA, AVG, AgNO_3_, or 1-MCP (same concentrations as in **Figure 2**) in the absence (Control) or presence of GR24, CTX, or both for 3h **(B)**. Epidermal strips of WT (Col-0) and mutants *etr1-1*, *etr1–3* and *eto1–1* were incubated in MES buffer alone (Control), or containing GR24, CTX, or both for 3 h **(C)**. Epidermal strips of WT (Ws) were incubated in MES buffer alone, or containing PTX in the absence (Control) or presence of GR24, ACC, or both for 3 h, and epidermal strips of mutants *gpa1-1*, *gpa1-2*, *cGα1* and w*Gα1* were incubated in MES buffer alone, or containing GR24, ACC, or both for 3 h After treatment, stomatal apertures were recorded **(A–C)**. **(D)** Leaves of WT (Ws) were incubated in MES buffer alone, or containing PTX without or with GR24 for 3 h, and leaves of of mutants *gpa1–1* and *gpa1–2* were incubated in MES buffer alone, or containing GR24 for 3 h The treated leaves were enclosed in a vial for another 3 h, and ethylene was collected and quantified **(D)**. The Data in **(A–D)** are represented as means ± SEs of three replicates [n = 90 for **(A–C)**; n = 3 for **(D)**]. Means with different letters are significantly different at *P* < 0.05.

Both ethylene and Gα activate H_2_O_2_ production and H_2_S generation, suggesting they operate within a shared signaling pathway. To declare their relationship, we performed complementation experiments. CTX effectively restored GR24-induced stomatal closure in wild-type (Col-0) plants treated with AOA, AVG, AgNO_3_, or 1-MCP, as well as in mutants *etr1–1* and *etr1-3* (**Figures 8A, B**). In contrast, ACC failed to rescue the stomatal closure defect in PTX-treated wild-type (Ws) plants or in *gpa1–1* and *gpa1–2* mutants (**Figure 8C**). These results indicated that Gα acted downstream of ethylene in SL-induced stomatal closure. To further validate the conclusion, we examined whether Gα affected ethylene production. GR24 induced ethylene synthesis in PTX-treated wild-type plants and in *gpa1–1* and *gpa1–2* mutants (**Figure 8D**). Combined with the results of **Figures 8A–C**, the data further consolidated the conclusion that ethylene acted upstream of Gα to mediate SL-induced stomatal closure in *Arabidopsis*.

## Discussion

### SL induced stomatal closure in *Arabidopsis*

SL play diverse roles in plant growth and development, and appear to integrate multiple signaling pathways to enhance plant adaptation to environmental stress (Dong et al., 2024, 2025). For example, SL can regulate root development, secondary growth, and leaf senescence (Rasmussen et al., 2012; Agusti et al., 2011; Ueda and Kusaba, 2015). Mutants impaired in SL biosynthesis or signaling exhibit heightened sensitivity to drought, salinity, and osmotic stress, highlighting the importance of SL in stress acclimation (Ha et al., 2014; Liu et al., 2015). Moreover, SL negatively regulate vessel element formation (Zhao et al., 2025) and can enhance drought resistance in cotton (Dong et al., 2024, 2025). SL also influence stomatal behavior, as evidenced by the more opening stomata and elevated conductance in *max2* mutants (Liu et al., 2015; Piisilä et al., 2015; Lv et al., 2018). The underlying mechanism involves SL-triggered stomatal closure, which depends on H_2_O_2_ synthesis, NO production, and SLAC1 activation via an ABA-independent pathway (Lv et al., 2018). These findings collectively suggest that manipulating SL biosynthesis or signaling could offer a viable strategy for improving plant stress adaptation. Correspondingly, exogenous application of the SL analog GR24 enhances drought tolerance in *Arabidopsis* (Ha et al., 2014), reinforcing the potential utility of SL-based compounds in boosting resilience to abiotic stress. Nevertheless, the precise perception and signaling mechanisms of SL in guard cells remain poorly defined. In this study, we demonstrate that SL induces stomatal closure in *Arabidopsis*. The reversible stomatal closure triggered by GR24 in wild-type plants further confirms the promoting role of SL in this process (**Figures 1A, B**), which is coincident with the existing literature (Lv et al., 2018; Ma et al., 2024). These results further support the role of SL-related pathways in plant stress adaptation and their prospective application for enhancing abiotic stress tolerance.

### Ethylene and Gα are required for stomatal closure by SL

SL cooperates with other phytohormones such as ABA, auxin, and cytokinin to regulate abiotic stress responses (Bhoi et al., 2021). Ethylene, which modulates various plant physiological processes (Kendrick and Chang, 2008), often functions through complex hormonal interactions (Wang and Irving, 2011). In cotton, ethylene acts downstream of BR signaling during fiber elongation and stomatal movement (Shi et al., 2006, 2015), while BR signaling components also participate in ethylene-mediated growth and defense responses (Deslauriers and Larsen, 2010). Ethylene is known to induce stomatal closure (Desikan et al., 2006). G proteins, which are critical for transducing extracellular signals into cellular responses, additionally regulate guard cell responses to ABA, extracellular calmodulin, BR, and extracellular ATP (Wang et al., 2001; Li et al., 2009; Hao et al., 2012; Shi et al., 2015). Notably, both ethylene biosynthesis and Gα activation have been implicated in BR-induced stomatal closure (Shi et al., 2015). However, whether ethylene and G proteins contribute to SL-induced stomatal movement remains unknown. In this study, inhibitors of ethylene synthesis or perception, as well as the Gα inhibitor PTX, significantly attenuated GR24-induced stomatal closure (**Figures 2**, **3**). Conversely, the ethylene precursor ACC and the Gα activator CTX elicited stomatal closure similar to GR24 in the wild type (**Figures 3**, **4**). GR24 also promoted ethylene production in wild-type plants (**Figure 2**). These results indicate that ethylene biosynthesis and Gα activation are both necessary for SL-induced stomatal closure. GR24 effectively closed stomata in *eto1-1*, *cGα1* and *wGα1* mutants, its effects were abolished in *etr1-1*, *etr1-3*, *gpa1–1* and *gpa1–2* mutants (**Figures 2**, **3**), and GR24 upregulated transcript levels of ethylene biosynthesis genes (*ACS* family) and the Gα subunit gene *GPA1* (**Figures 2**, **3**), reinforcing the involvement of ethylene and Gα in SL-triggered stomatal closure in *Arabidopsis*. Collectively, these results demonstrate that both ethylene and Gα are required for SL-induced stomatal closure. Nevertheless, the mechanism by which SL stimulate ethylene synthesis, as well as the roles of other G protein subunits in SL guard cell signaling, remains to be elucidated.

### Ethylene activates H_2_O_2_ and H_2_S synthesis in stomatal closure by SL

Previous studies have shown that ethylene induces stomatal closure via H_2_O_2_ production (Desikan et al., 2006). Further evidence indicates that H_2_O_2_ also mediates ethylene-dependent stomatal closure in response to UV-B (He et al., 2011), and that ethylene contributes to BR-induced guard cell signaling through Gα-triggeed H_2_O_2_ and NO generation (Shi et al., 2015). Additionally, H_2_S produced by L-/D-CDes is involved in ethylene-induced stomatal movement (Liu et al., 2011; Hou et al., 2013) and also participates in melatonin-induced stomatal closure (Wang et al., 2023). Although H_2_O_2_-dependent H_2_S production has been linked to SL-induced stomatal closure (Ma et al., 2024), whether ethylene acts upstream of H_2_O_2_ and H_2_S in SL signaling remains unknown. Our results demonstrated that ethylene indeed induced both H_2_O_2_ and H_2_S synthesis in SL-induced stomatal closure. Inhibitors of ethylene synthesis or perception reduced GR24-triggered H_2_O_2_ and H_2_S biosynthesis, as well as L-/D-CDes activity in wild-type plants. These responses were enhanced in *eto1-1* mutant, but absent in *etr1–1* and *etr1–3* mutants (**Figures 4**, **5**). Furthermore, ACC-induced stomatal closure, H_2_O_2_ accumulation, H_2_S biosynthesis, and L-/D-CDes activity increase were abolished in *AtrbohD*, *AtrbohF* and *AtrbohD/F* mutants, as well as in *Atl-cdes* and *Atd-cdes* mutants (**Figures 4**, **5**). Taken together, these findings establish that ethylene mediates SL-induced stomatal closure through H_2_O_2_ production and subsequent H_2_S biosynthesis. This conclusion is further supported by the ability of exogenous H_2_O_2_ and NaHS to restore GR24-induced stomatal closure in *etr1* mutants (**Figures 4**, **5**), confirming that ethylene functions upstream of both H_2_O_2_ and H_2_S in the SL-triggered stomatal closure in *Arabidopsis*. These results confirm that ethylene promotes H_2_O_2_ and H_2_S production during SL-induced guard cell signaling. However, the mechanism by which ethylene mediates the stimulatory effect of SL on H_2_O_2_ and H_2_S generation remains unclear.

### Gα activates H_2_O_2_ and H_2_S synthesis in SL-induced stomatal closure

In plants, G proteins play critical roles in numerous biological processes (Tsugama et al., 2013; Zhang et al., 2021), and they are also key regulators in plant adaptation to environmental stresses, including salinity and nutrient deprivation (Yadav et al., 2024; Cho, 2024). Previous studies have established that Gα-dependent H_2_O_2_ production contributes to stomatal closure triggered by various factors, such as extracellular calmodulin, extracellular ATP, UV-B radiation, ethylene, and BR (Chen et al., 2004; Zhang et al., 2011; Hao et al., 2012; He et al., 2013; Ge et al., 2015; Shi et al., 2015). Both H_2_O_2_ and H_2_S biosynthesis are involved in stomatal closure induced by ethylene (Hou et al., 2013), salt stress (Ma et al., 2019b), and CdCl_2_ (Ma et al., 2019a), and BR (Ma et al., 2022). Furthermore, Ma et al. (2024) indicated that H_2_O_2_ acts upstream of H_2_S synthesis during stomatal closure induced by SL. Nevertheless, it remained unclear whether Gα mediates SL-induced stomatal closure through H_2_O_2_ production and H_2_S synthesis. Our study reveals that in *Arabidopsis*, Gα-activated H_2_O_2_ production and H_2_S biosynthesis together mediate stomatal closure in response to SL. In wild-type plants, Gα inhibitor PTX markedly reduced GR24-triggered H_2_O_2_ accumulation in guard cells, as well as the increase in H_2_S biosynthesis and L-/D-CDes enzyme activity. These GR24-elicited responses were maintained in the *cGα1* and *wGα1* mutants but were abolished in *gpa1–1* and *gpa1–2* mutants (**Figures 6**, **7**). Additionally, in the wild type, stomatal closure induced by CTX, along with the associated guard cell H_2_O_2_ accumulation, H_2_S biosynthesis, and enhanced L-/D-CDes activity, was eliminated in *AtrbohD*, *AtrbohF*, *AtrbohD/F* mutants, as well as in *Atl-cdes* and *Atd-cdes* mutants, respectively (**Figures 6**, **7**). These results demonstrate that Gα functions via both H_2_O_2_ production and H_2_S biosynthesis. Finally, applying exogenous H_2_O_2_ or the H_2_S donor NaHS restored GR24-induced stomatal closure in *gpa1–1* and *gpa1–2* mutants (**Figures 6**, **7**), confirming that Gα acts upstream of H_2_O_2_ and H_2_S generation in *Arabidopsis*. Collectively, these findings provide strong evidence that Gα promotes the production of H_2_O_2_ and H_2_S in SL-initiated guard cell signaling. However, the mechanism by which Gα mediates the enhancing effect of SL on H_2_O_2_ and H_2_S biosynthesis requires further investigation.

### Ethylene acts upstream of the G protein α-subunit in SL-induced stomatal closure

Heterotrimeric G proteins serve as crucial signaling molecules in eukaryotes. In the model plant *A. thaliana*, this pathway is represented by a minimal set of subunits: one Gα gene (*GPA1*), one Gβ gene (*AGB1*), and three Gγ genes (*AGG1-AGG3*) (Jones and Assmann, 2004; Chakravorty et al., 2011). These protein complexes modulate a wide range of physiological functions, such as development, hormonal signaling, and adaptation to stress (Okamoto et al., 2001, 2009; Shi et al., 2015; Zhang et al., 2021). Interactions exist between ethylene signaling and the G protein pathway. Specifically, Gα subunit has been shown to participate in ethylene responses. For example, Gα mediates ethylene-induced triple response (Ullah et al., 2002) and contributes to ethylene-mediated inhibition of root growth (Wang et al., 2006). Notably, ethylene facilitates BR-induced stomatal movement by activating Gα (Shi et al., 2015). Furthermore, G proteins function as negative regulators of ABA responses (Xu et al., 2024), while ethylene antagonizes ABA-induced stomatal closure through the upregulation of negative regulators ABI1 and ABI2 in the ABA pathway (An et al., 2025). However, a potential role for G proteins in ethylene signaling during SL-induced stomatal closure remains unknown. Our results proved that both ethylene and Gα activated H_2_O_2_ production and H_2_S biosynthesis, suggesting they operate within a shared signaling cascade (**Figure 4**-**7**). CTX restored GR24-induced stomatal closure in wild-type plants treated with inhibitors of ethylene biosynthesis or perception, as well as in *etr1–1* and *etr1–3* mutants. Conversely, ACC failed to rescue the stomatal closure defect in PTX-treated wild-type plants or in *gpa1–1* and *gpa1–2* mutants (**Figure 8**). These results suggest that Gα acts downstream of ethylene in SL-induced stomatal closure. Additionally, GR24 significantly increased ethylene production in PTX-treated wild-type plants and in *gpa1–1* and *gpa1–2* mutants (**Figure 8**), further supporting the model that ethylene promotes stomatal closure by activating Gα in SL-induced stomatal closure in *Arabidopsis*. While our study underscores the essential role of Gα in ethylene-triggered guard cell signaling, future investigations are needed to determine whether other G protein subunits are involved in SL responses within guard cells across different plant species.

## Conclusions

**Figure 9:**
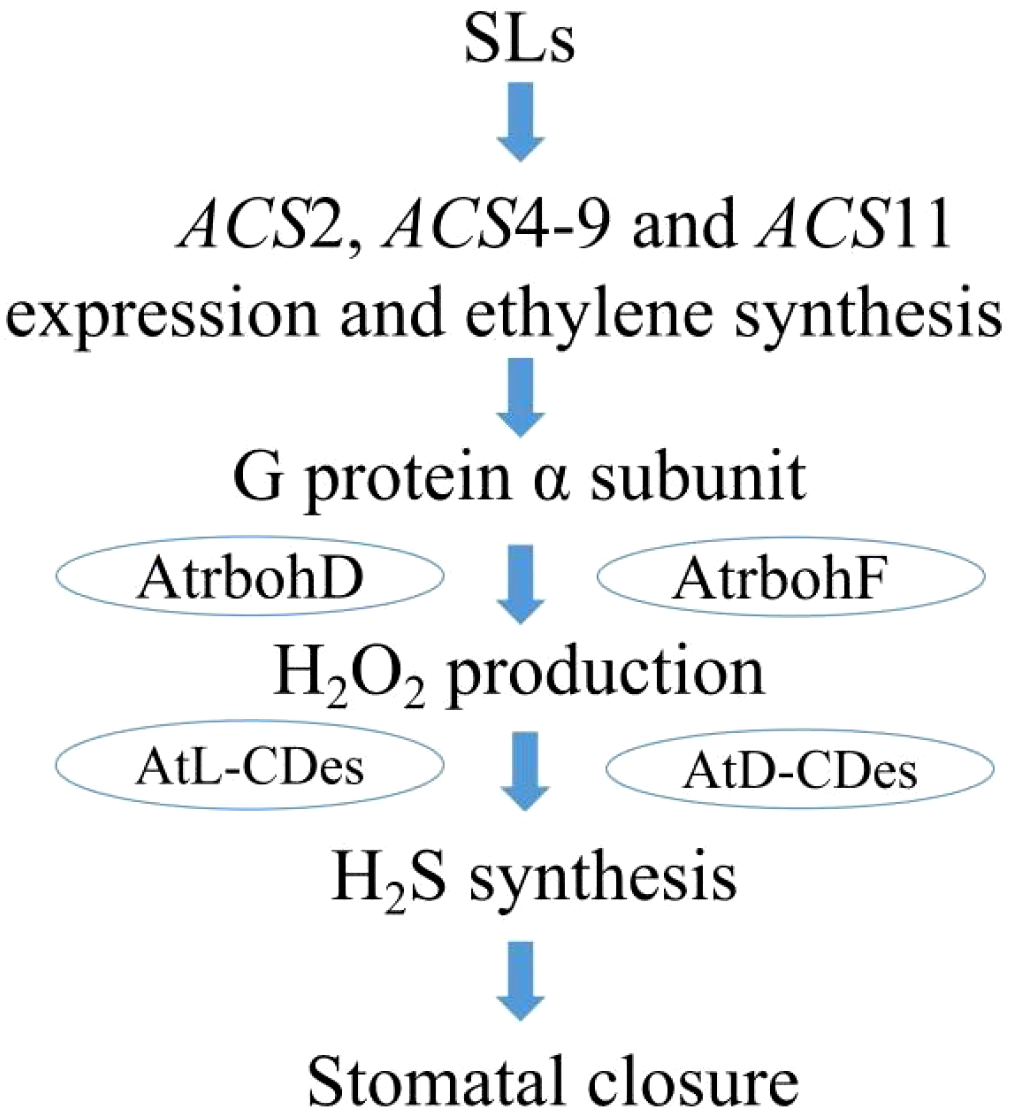
Model showing the possible signaling pathway for SL-induced stomatal closure in *Arabidopsis*. GR24 induces ethylene synthesis, which activates G protein, thereby inducing H_2_O_2_ production by AtrbohD and AtrbohF, and then causes H_2_S synthesis catalyzed by AtL-CDes-/AtD-CDes, finally leading to stomatal closure.

Our prior research demonstrated that H_2_O_2_ acts upstream of H_2_S in SL-triggered stomatal closure in *Arabidopsis* (Ma et al., 2024). Integrating these earlier results with the present data, we propose a signaling pathway for SL-triggered stomatal closure (**Figure 9**). In this model, SL upregulated ACS expression and promoted ethylene biosynthesis, which in turn activated Gα. The activated Gα then stimulated H_2_O_2_ production primarily through AtrbohD and AtrbohF, which triggers H_2_S synthesis catalyzed by AtL-CDes and AtD-CDes, ultimately leading to stomatal closure. These findings not only reinforce the function of SL in modulating stomatal movement but also elucidate the specific contributions of ethylene and G proteins to guard cell SL signaling, thereby deepening our mechanistic understanding of SL-regulated stomatal closure. However, several important questions remain: (1) the mechanism by which SL upregulate *ACS* expression and enhance ethylene production is still unclear; (2) the precise process through which activated Gα initiates H_2_O_2_ and H_2_S biosynthesis requires further exploration; and (3) the potential involvement of protein kinases and Ca^2+^ in SL-induced stomatal responses, as well as their interplay with ethylene, H_2_O_2_, and H_2_S, needs further elucidation.

## Data Availability

The original contributions presented in the study are included in the article/supplementary material. Further inquiries can be directed to the corresponding author.
